# Protection of Melanized *Cryptococcus neoformans* from Lethal Dose Gamma Irradiation Involves Changes in Melanin's Chemical Structure and Paramagnetism

**DOI:** 10.1371/journal.pone.0025092

**Published:** 2011-09-22

**Authors:** Abdelahad Khajo, Ruth A. Bryan, Matthew Friedman, Richard M. Burger, Yan Levitsky, Arturo Casadevall, Richard S. Magliozzo, Ekaterina Dadachova

**Affiliations:** 1 Department of Chemistry, Brooklyn College of the City University of New York, Brooklyn, New York, United States of America; 2 Departments of Chemistry and Biochemistry, The Graduate Center of the City University of New York, New York, New York, United States of America; 3 Department of Nuclear Medicine, Albert Einstein College of Medicine of Yeshiva University, Bronx, New York, United States of America; 4 Department of Microbiology and Immunology, Albert Einstein College of Medicine of Yeshiva University, Bronx, New York, United States of America; University of Wisconsin – Madison, United States of America

## Abstract

Certain fungi thrive in highly radioactive environments including the defunct Chernobyl nuclear reactor. *Cryptococcus neoformans* (*C. neoformans*), which uses L-3,4-dihydroxyphenylalanine (L-DOPA) to produce melanin, was used here to investigate how gamma radiation under aqueous aerobic conditions affects the properties of melanin, with the aim of gaining insight into its radioprotective role. Exposure of melanized fungal cell in aqueous suspensions to doses of γ-radiation capable of killing 50 to 80% of the cells did not lead to a detectable loss of melanin integrity according to EPR spectra of melanin radicals. Moreover, upon UV-visible (Xe-lamp) illumination of melanized cells, the increase in radical population was unchanged after γ-irradiation. Gamma-irradiation of frozen cell suspensions and storage of samples for several days at 77 K however, produced melanin modification noted by a reduced radical population and reduced photoresponse. More direct evidence for structural modification of melanin came from the detection of soluble products with absorbance maxima near 260 nm in supernatants collected after γ-irradiation of cells and cell-free melanin. These products, which include thiobarbituric acid (TBA)-reactive aldehydes, were also generated by Fenton reagent treatment of cells and cell-free melanin. In an assay of melanin integrity based on the metal (Bi^+3^) binding capacity of cells, no detectable loss in binding was detected after γ-irradiation. Our results show that melanin in *C. neoformans* cells is susceptible to some damage by hydroxyl radical formed in lethal radioactive aqueous environments and serves a protective role in melanized fungi that involves sacrificial breakdown.

## Introduction

**Figure 1:**
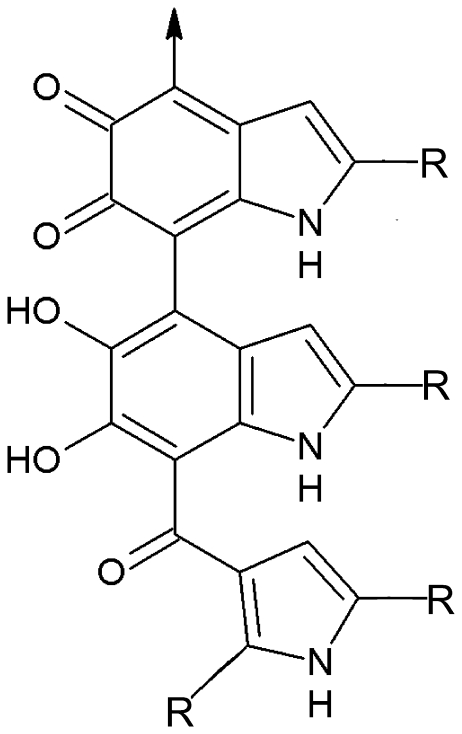
Structure of eumelanin oligomer, adapted from [**11**]. R = H, COOH, or other subunits.

Melanins are complex polymers, formed by oxidation of various precursors, including tyrosine, tryptophan, and L-3,4-dihydroxyphenylalanine (L-DOPA) and are found in cells of all biological kingdoms. Figure 1 shows an accepted structure for the subunits of eumelanin produced from L-DOPA. In microorganisms, melanin protects from damage by UVC light [1], [2], reduces oxidative stress [3], and participates in energy transduction and electron transfer processes [4]–[7]. Recent evidence from one of our laboratories suggested that melanin in live *Cryptococcus neoformans* (*C. neoformans*) cells can function both in energy transduction [8] and as a radioprotectant [9]. This fungus is of special interest because melanized microbial species are found in highly radioactive environments such as the cooling pools of nuclear reactors, in the stratosphere, in space stations, and inside the damaged nuclear reactor at Chernobyl (reviewed in [10]). The physics of interaction of ionizing radiation with synthetic eumelanins and pheomelanins (sulfur-containing) was described based on a variety of physico-chemical techniques including electron paramagnetic resonance (EPR) spectroscopy [9], [11]. Dry melanins were reported to be resistant to high-dose (300 Gy), high-energy (^137^Cs, 661.6 keV) irradiation because of an unchanged EPR signal characteristic of stable melanin radicals [12]. Other studies have reported that ionizing radiation could damage melanin [13], [14], but no specific chemical analyses were presented, and this is among the gaps in knowledge we aimed to address here.

In this work, the effects of lethal doses of ionizing radiation on viable fungal cells were investigated under aerobic aqueous conditions. But which properties of melanin are the most meaningful to probe to demonstrate resistance to, or damage from, the effects of ionizing radiation? While radicals in melanin are among the intriguing features of its structure, the relationship between these radicals and functional properties of the polymer in living cells is not clearly understood. The radical population is known to depend on the position of the equilibrium between reduced and oxidized quinoid groups (Eq. 1),
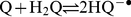
(1)which shifts in response to pH, temperature, light, and other effectors [15], [16] (Q denotes *o*-quinone, H_2_Q is *o*-hydroquinone (quinol), and HQ^−**•**^ is an EPR detectable *o*-semiquinone radical). Melanin also interacts with metal ions, which can bind to *o*-semiquinone moieties in the polymer and shift the equilibrium [17] though the specific relevance of this property, and that of other effectors, to fungal physiology have not been established.

While much is unknown about the relationship between melanin's properties and its cellular function(s), there is convincing evidence that it serves as a radioprotective agent [9]. Therefore, it was considered important to investigate the effects of ionizing radiation on cellular melanin in aqueous environments. EPR spectroscopy, metal ion binding, and chemical analysis techniques were used to study the response of melanized *C. neoformans* cells to γ radiation. Among the key findings is that chemical modification of melanin could be detected in samples that showed an actual increase in melanin radical population after γ-irradiation. Thiobarbituric acid (TBA)-reactive aldehydes were released due to hydroxyl radical-induced modification of melanin. This novel observation may have implications in the physiology of melanized fungi like *C. neoformans* and even the physiology of other cell types.

## Materials and Methods

### *Cryptococcus neoformans* cell growth

The strain used for these experiments was cap67, an acapsular, avirulent strain of *C. neoformans* derived from the serotype D strain B3501 [18]. This strain was used because it is safe to handle and because γ radiation would remove capsular material from wild-type cells [19] and thereby confound our analyses. The cells were grown for 20 days in minimal medium (29.4 mM KH_2_PO_4_, 10 mM MgSO_4_, 13 mM glycine, 15 mM D-glucose, 3 µM thiamine) [20] supplemented or not with 1 mM L-DOPA, in the dark at room temperature (23°C), shaking at 150 rpm. Only those cells grown in the presence of L-DOPA become melanized; thus, cells grown in the absence of L-DOPA provide minimally perturbed control cells.

The cells were harvested by diluting the cultures with phosphate-buffered saline (PBS) pH 5.9 to improve pelleting, followed by centrifugation at 1300**×** g for 10 min, then washing twice with PBS. The cells were resuspended in PBS and the cell concentration was adjusted to approximately 2**×** 10^9^ per mL. Melanized-cell ghosts, which are shells composed of essentially only melanin, were prepared from melanized *C. neoformans* cells as previously described [21].

### Electron paramagnetic resonance spectroscopy of untreated *C. neoformans* cells

Suspensions of freshly harvested *C. neoformans* cells (1.9**×**10^9^ melanized cells per mL or 1.2**×**10^9^ non-melanized cells per mL) were placed in 4 mm precision-bore quartz EPR tubes and were frozen by immersion in liquid nitrogen immediately after filling the tubes. EPR spectra were recorded at 77 K using a Bruker E500 ElexSys EPR spectrometer operating at X-band with an ER4122SHQE resonator cavity in which samples were held in a quartz immersion finger Dewar filled with liquid nitrogen. Data acquisition and manipulation was performed using *XeprView* and *WinEPR* software (Bruker). Experimental parameters used to acquire the EPR spectra were as follows: modulation amplitude, 1 G; microwave power, 0.1 mW; modulation frequency, 100 kHz; microwave frequency, 9.49 GHz; scan rate, 1.2 G/s; conversion time, 163 ms; time constant, 1310 ms; number of scans averaged, 3. All spectra were obtained under identical instrumental conditions.

### Xenon-lamp illumination of *C. neoformans* cells

Illumination of cell suspensions was carried out using a 75-watt xenon lamp (L2194-02, Hamamatsu Photonics, Japan). Light was focused on the samples in quartz EPR tubes (∼0.8 cm^2^ cross-section) at room temperature. Each sample received ∼7 microeinsteins per second for the desired illumination times. For 77 K illumination, frozen samples in quartz EPR tubes were held in a liquid nitrogen-filled quartz finger Dewar.

### Gamma-irradiation and Fenton-reagent treatment of *C. neoformans* cells and cell-free melanins

Cell suspensions (6.5**×**10^9^ melanized cells/mL and 5.5**×**10^9^ non-melanized cells/mL), melanized-cell ghosts (2 mg/mL), and L-DOPA synthetic melanin (Sigma) (2 mg/mL), each in PBS, were γ-irradiated at room temperature in microcentrifuge tubes; frozen cell samples were irradiated in quartz EPR tubes suspended in liquid nitrogen. The radiation dose was 11.94 Gy/min, delivered from a cesium-137 source in a Mark I irradiator (JL Shepherd and Associates, San Fernando, CA). The doses delivered for 10 and 30 minutes, 120 and 360 Gy, respectively, are in a range lethal to 50–80% of the cell population [9]. Samples irradiated at room temperature were transferred to quartz EPR tubes and immediately frozen in liquid nitrogen. For control samples, microcentrifuge tubes or EPR tubes, filled with PBS alone, were similarly irradiated at room temperature or at 77 K. Gamma-irradiation of quartz EPR tubes generates paramagnetic centers [22], [23] and the background EPR spectrum (a narrow singlet with linewidth of ∼3.5 G at *g* = 2.0004) recorded from a tube filled with PBS and irradiated along with cell samples was subtracted from data where appropriate.

The Fenton reagent reactions were carried out using Fe(NH_4_)_2_(SO_4_)_2_ **•**6H_2_O and H_2_O_2_ in deionized water added to 800 uL of *C. neoformans* cells, melanized cell ghosts, or synthetic melanin each suspended in PBS at the concentrations given above, to give final Fe(II) and H_2_O_2_ concentrations of 5 mM and 10 mM respectively. Peroxide was added immediately after iron. Incubations were carried out at room temperature for 15 min, followed by centrifugation and recovery of supernatants.

### Absorption spectroscopy of supernatants

Supernatants were collected from γ-irradiated cells and other samples by centrifugation to remove suspended material and were diluted 10-fold with PBS. Spectra were recorded using an NT14 UV-Vis spectrophotometer (Aviv Associates, Lakewood, NJ) interfaced to a personal computer. Spectra of supernatants from cells were normalized to correct for differences in cell concentration.

Gamma-irradiated water or PBS collected from microcentrifuge tubes did not contain any detectable UV-absorbing material.

### Peroxidation methods

Peroxide digestion of cell-free melanins was carried out as in [12] and [24] by suspending 1 mg of melanized-cell ghosts or synthetic melanin in 1.0 mL of deionized water. K_2_CO_3_ and H_2_O_2_ were added to give final concentrations of 0.1 M and 0.12%, followed by incubation at 100°C for 20 min. After cooling of the reaction mixture, residual H_2_O_2_ was decomposed by adding 200 µl of 10% Na_2_SO_3_. The mixture was then acidified with 1.0 mL of dilute HCl. The resulting solution was centrifuged and the supernatant was used for optical measurements.

Pyrrole-2,3-dicarboxylic acid (PDCA) and pyrrole-2,3,5-tricarboxylic acid (PTCA) were synthesized from 5-hydroxyindole and 5-hydroxyindole-2-carboxylic acid (Sigma), respectively, as in [12] and [24]. For optical spectra, 100 µg/mL PDCA and PTCA were dissolved in deionized water.

### 2-thiobarbituric acid-reaction methods

The TBA reactions were performed according to [25] using 200 µL of supernatants recovered from irradiated samples or Fenton reagent-treated samples, added to 800 µL of 31 mM TBA solution containing 1 mM Na_2_EDTA in water, followed by incubation at 90°C for 20 min. PBS was similarly treated with TBA/EDTA solution to establish a blank spectrum for subtraction from that of TBA adducts formed from the irradiated samples. For background correction in the case of Fenton reagent-treated samples, a TBA-reaction blank was prepared using 200 µL of a mixture containing 5 mM Fe(II) and 10 mM H_2_O_2_ in PBS that had been incubated for one hour, centrifuged to remove precipitated iron, and then treated as above with 800 µL of 31 mM TBA containing 5 mM EDTA. Optical spectra of the colored TBA adducts were recorded immediately after the samples had cooled to room temperature. Malondialdehyde (MDA) used as a standard was prepared from commercial malonaldehyde bis(dimethylacetal) (Fisher) according to [26].

### Bismuth uptake by *C. neoformans* cells

Cap67 *C. neoformans* cells were grown in minimal medium with or without 1 mM L-DOPA for 20 days, washed, and resuspended in 1.8 mL PBS. Melanized cells (7.6**×**10^6^ cells in 1 mL) or non-melanized cells (1.0**×**10^7^ cells/mL) were irradiated in PBS with a dose of 13.88 Gy/min for 30 min. The ^225^Ac used to generate ^213^Bi was obtained from the Institute for Transuranium Elements, Heidelberg, Germany. The ^225^Ac-^213^Bi generator was constructed using MP-50 cation exchange resin, and ^213^Bi was eluted with 0.15 M HI as described in [27] and titrated to pH 5 with ammonium acetate. For each experiment, approximately 6 µCi of ^213^Bi were mixed with *C. neoformans* cells. Over the course of two hours, the cells were centrifuged, an aliquot of the supernatant was removed, and the radioactivity remaining in that portion of the supernatant was measured. The pellets were then resuspended and the incubation resumed with the remaining suspensions. The total radioactivity remaining in each sample was determined by measuring the radioactivity in an equal volume from a cell-free sample of ^213^Bi in PBS. Metal binding was calculated using the formula:
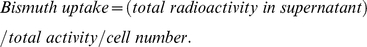



## Results

### EPR spectroscopy of *C. neoformans* cells

**Figure 2:**
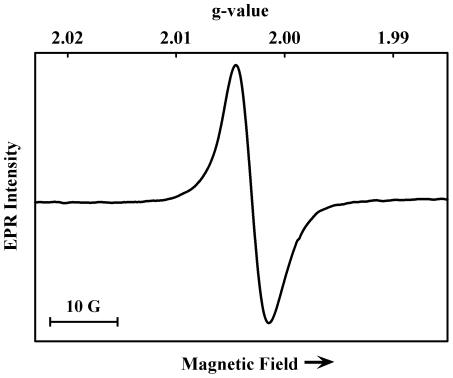
EPR spectrum (77 K) of melanized *C. neoformans* cells (1.9×10^9^ cells/mL).

All melanins contain a small population of semiquinone radicals within their structure and exhibit a characteristic EPR signal. Melanized *C. neoformans* cells collected from cultures grown in the presence of L-DOPA are black and exhibit an apparent singlet X-band EPR signal (77 K) at *g* = 2.0030 with a linewidth (peak to trough) of 5 Gauss (Fig. 2) similar to that of synthetic eumelanin [17], [28]. Comparison of the signal intensity to that from a suspension of melanized-cell ghosts suggests a melanin concentration of ∼3.0 mg/mL in typical cell samples. The radical concentration in melanins is known to respond to effectors including pH, temperature, and light, which alter the equilibrium between reduced and oxidized quinoid species [15], [16]. To investigate the properties of melanin in the cells and for later experiments in which the effects of γ-irradiation were monitored, the EPR signal from cell suspensions was recorded before and after illumination with high intensity light from a Xe lamp. This approach provides a probe of the integrity of melanin subunit structure, as it depends on the formation of new semiquinone radicals different from the intrinsic radicals.

**Figure 3:**
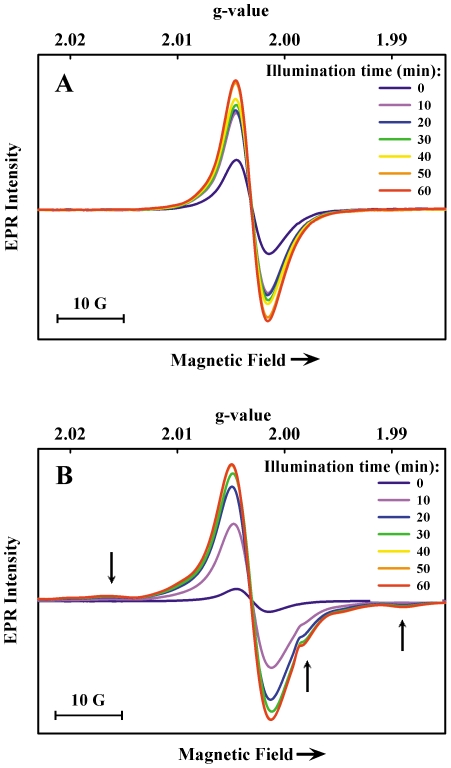
EPR spectra (77 K) of melanized *C. neoformans* cells after Xe-lamp illumination. A) cells were illuminated at room temperature for the time periods indicated and frozen immediately after removal from the light source; B) cells were frozen after collection from cultures and were illuminated at 77 K for the time periods indicated.

Illumination of cells at room temperature caused an increase in EPR signal intensity (measured at 77 K) in a dose-dependent manner, reaching a maximum of 2.5-fold after ∼60 min (Fig. 3A). Illumination of frozen cell suspensions produced a 10-fold maximum increase in intensity after ∼60 min (Fig. 3B). Samples stored in the dark at 77 K retained the increased intensity for at least several days after illumination (not shown). In general, the response to light is consistent with the formation of semiquinones that persist briefly after illumination at room temperature, but which are trapped at 77 K. A small broadening of the signal (less than 1 Gauss) was also noted as previously reported [29] suggesting that the new radical sites are structurally distinct from the intrinsic radical sites. Non-melanized cell samples, which were pale in color, did not exhibit an EPR signal related to the typical melanin signal before or after Xe-lamp illumination (not shown).

In addition to the melanin radicals produced photochemically, illumination at 77 K produced other dilute paramagnetic species (arrows, Fig. 3B) in samples of both melanized and non-melanized cells. These features arise from photo-induced cellular radicals and paramagnetic centers in illuminated quartz (shoulder at *g* ∼1.999) [23]. No difference was observed between melanized and non-melanized cells in these background signals.

**Figure 4:**
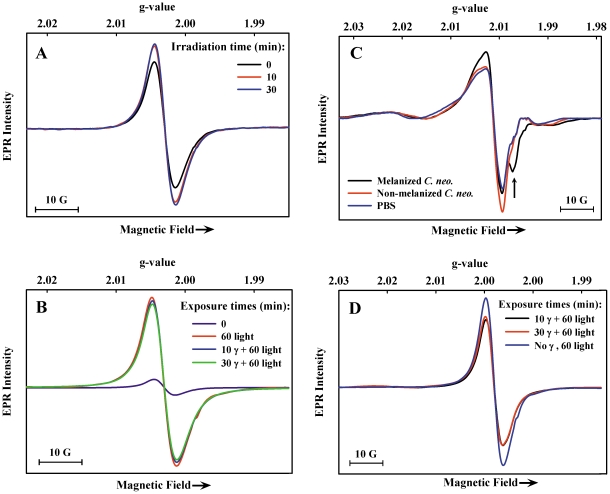
EPR spectra (77 K) of γ-irradiated and Xe-lamp illuminated *C. neoformans* cells. A) melanized cells γ-irradiated at room temperature (11.94 Gy/min), then frozen in liquid nitrogen; B) γ-irradiated melanized cells from (A) illuminated for the indicated time periods after freezing at 77 K; C) γ-irradiated (11.94 Gy/min) frozen (77 K) melanized cells, non-melanized cells, and PBS; D) γ-irradiated melanized cells from (C) stored frozen for 2 weeks then illuminated at 77 K for the indicated time periods.

The above survey of melanin radical behavior in whole *C. neoformans* cells provided the basis for interpretation of the effects of γ-irradiation. Cell suspensions were irradiated with two different doses of γ rays (10 and 30 min, ∼120 and 360 Gy total, capable of killing about 50 and 80% of cells, respectively [9]) followed by freezing in liquid nitrogen within 1 min of removal from the beam. These radiation doses are in a range known from prior work to demonstrate significant protection of melanized *C. neoformans* cells relative to non-melanized cells [9]. Here, the γ-irradiation caused a 30% increase in melanin radical signal intensity measured at 77 K for the higher dose (Fig. 4A). This gain in intensity, which could arise from a number of different processes, was reversed upon thawing and incubating the samples at room temperature (not shown) and was not explored further. In non-melanized cell samples, no EPR signal related to the typical melanin signal was detected after γ-irradiation (not shown).

To probe for changes in the photoresponse of melanin, γ-irradiated melanized cell samples were subjected to illumination at 77 K as above. The EPR signal intensity again increased approximately 10-fold (Fig. 4B) compared to the intensity increase before irradiation (Fig. 3B) suggesting that no loss in melanin integrity had occurred.

To test for rapidly reversible changes occurring at room temperature that escaped detection in the EPR samples described above (which were frozen after removal from the radiation beam), melanized cells were γ-irradiated at 77 K. The observations here were complicated by new EPR signals that overlapped with and nearly completely masked the melanin radical signal, which is indicated by the arrow in Fig. 4C. The broad new signals arise from hydroxyl and hydroperoxyl radicals produced in γ-irradiated ice, consistent with water radiolysis as their principal source [30]–[33]. These signals, which slowly decay by radical recombination [32] upon storage at 77 K or quickly decay at room temperature, were also seen with nearly equal intensities for γ-irradiated non-melanized cell suspensions and frozen PBS. Any of these new radicals for which EPR signals were detected, as well as other short-lived species produced during radiolysis of water such as e^−^_aq_, CO_2_^−**•**^, O_2_^−**•**^, H**^•^** [13], [31] could contribute to damage to cells and melanin in our experiments and in environments where ionizing radiation is present.

In order to examine melanin radical signals in the samples that had been γ-irradiated, the non-melanin signals were allowed to decay by thawing the sample for 1 hr. The EPR spectrum after refreezing showed an intensity of the melanin radical signal was approximately 80% of its value before γ-irradiation (not shown). To extend this analysis, Xe-lamp illumination was applied; a lower photoresponse was observed for the two γ-irradiated samples, compared with the 10-fold increase for the non-irradiated sample (Fig. 4D). The small losses in the intrinsic radical population and in the photoresponse suggest that some damage had occurred to melanin, which may also have occurred in the room temperature protocol but was not detected by EPR. Therefore, a search for small molecule products was undertaken.

### Soluble products released from γ-irradiated *C. neoformans* cells, melanized-cell ghosts, and synthetic melanin

**Figure 5:**
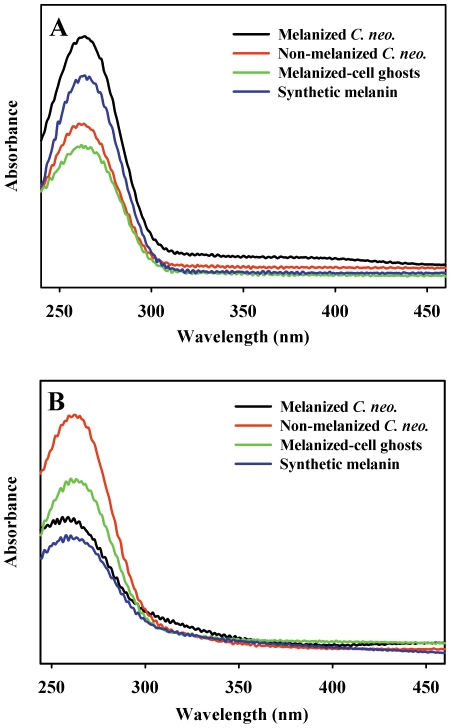
Optical spectra of supernatants collected from γ-irradiated or Fenton reagent-treated samples. A) melanized *C. neoformans* cells, non-melanized *C. neoformans* cells, melanized-cell ghosts, and synthetic melanin, irradiated for 30 min at room temperature in PBS; B) as in A, incubated with Fe(II) and H_2_O_2_ (Fenton reagent).

 An earlier report showed that radiolysis of melanin caused small changes in its broad and featureless optical absorption spectrum, but those observations did not provide specific structural insights [13]. If γ-irradiation causes chemical changes in melanin, soluble products might be found post irradiation. In fact, supernatants collected from γ-irradiated melanized (and non-melanized) *C. neoformans* cells, melanized-cell ghosts, and synthetic melanin all contained products exhibiting an absorption maximum near 263 nm (Fig. 5A). This observation provided evidence that damage to melanin had occurred and the soluble products have a UV-absorbing functional group in common with other cellular breakdown products.

Reproducibility in the γ-irradiation protocols was inadequate for rigorous calculation of yields of these UV-absorbing species. Furthermore, normalization of results for the cell-free heterogeneous samples is not feasible. Supernatants collected from unirradiated cells and cell-free melanin had negligible absorbance in the UV region (not shown).

Given that the γ-irradiated samples contained peroxyl radical detected by EPR and that melanin is digested by hydrogen peroxide [12], the soluble products in supernatants might have included the known principal peroxidation products PTCA or PDCA [12]. However, the optical spectra of the supernatants were different from those of authentic PTCA and PDCA, and different from ghost or synthetic melanin peroxidation products (which were most consistent with PDCA) (in Supplemental Information, Fig. S1-A,B) [34]. Thin layer chromatography of supernatants collected from γ-irradiated melanized cells did not reveal detectable amounts of PDCA or PTCA (not shown).

 Hydroxyl radical is among the abundant and potentially damaging species to which γ-irradiated cells were exposed. Therefore, an attempt to generate the soluble products described above was made using Fenton reaction chemistry (**Fe(II)+H_2_O_2_→Fe(III)+OH^−^+OH^•^**). Melanized and non-melanized cells and two sources of cell-free melanin were briefly incubated with Fenton reagents. The spectra of UV-absorbing material in supernatants collected from these samples (Fig. 5B) were similar to those from γ-irradiated samples, having λ_max_∼263 nm (Fig. 5A). The yields increased with increasing concentration of iron and hydrogen peroxide (not shown). These results strongly suggest that the soluble products generated by γ-irradiation of melanized cells include some from hydroxyl radical damage to melanin, since OH**^•^** is the reactive species common to both the γ-irradiation and the Fenton reagent protocols.

### Characterization of UV-absorbing products

**Figure 6:**
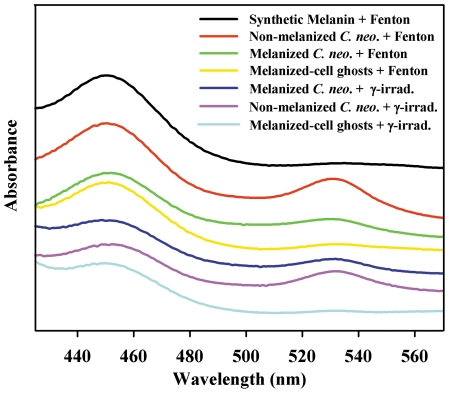
Optical spectra of TBA-adducts produced from supernatants collected from γ-irradiated samples. Spectra are offset for presentation.

 Malondialdehyde (MDA) is among the expected soluble products from γ-irradiation of cells along with other TBA-reactive substances arising from damage to biomolecules [26], [35], [36]. No report of such products coming from melanin, however, could be found. Supernatants from each γ-irradiated and Fenton-reagent treated sample were tested for TBA reactivity, which is a widely-used method for determination of small molecule aldehydes [26], [35]. A yellow chromophore (λ_max_ = 450 nm) was detected in all cases along with varying yields of another species with a λ_max_ = 532 nm typical of the MDA-TBA chromophore [26] (Fig. 6). The chromophore with a λ_max_ = 450 nm has been reported for TBA adducts of saturated and unsaturated aldehydes [36]–[39].

The TBA-adducts produced from supernatants collected from γ-irradiated and Fenton-treated melanized-cell samples were very similar (Fig. 6). For example, assuming that these adducts only produce TBA chromophores absorbing at 450 nm and/or 532 nm, a ratio of absorbances at these wavelengths should be constant for a similar aldehyde product profile. The A_450_/A_532_ was approximately 3.5 for melanized cells exposed to radiation or Fenton reagents. A lower ratio (2.4) for the non-melanized cell products is consistent with an absence of melanin-derived aldehydes. Interestingly, the chromophores generated using supernatants from Fenton reagent-treated cell-free melanins had optical ratios of 12 or 16, for ghosts or synthetic melanin respectively. These observations confirm that the aldehydes derived from melanin decomposition principally produce a TBA-adduct chromophore with a λ_max_ = 450 nm.

### Metal binding (bismuth uptake) to *C. neoformans* cells

The functional groups required for the intrinsic metal binding capacity of melanin [40] could also be a target of radiation damage in melanized cells, which was tested here using a ^213^Bi binding assay [41]. For unirradiated cells, the uptake of Bi^+3^ was ∼20% greater in melanized than in non-melanized cells (Table 1) and γ radiation had only limited effects. More abundant metal binding sites in melanized cells likely include carboxylates and other functional groups previously defined as divalent and trivalent metal ligands [15]. Importantly, no large change in metal-binding capacity was caused by γ-irradiation of melanized cells.

**Table 1:** ^213^Bi binding to *C. neoformans* cells.

	Melanized *C. neoformans*		Non-melanized *C. neoformans*	
	Unirradiated	γ-irradiated	Unirradiated	γ-irradiated
**Experiment 1** (n = 4)	85.8^a^±8.0	90.1±2.3	70.1±0.4	70.1±1.8
**Experiment 2** (n = 8)	78.9±5.8	85.0±3.8	64.5±3.1	67.2±1.6

(a) Expressed as a fraction of total radioactivity, normalized to cell count, ± standard deviation.

## Discussion

The nature of the interaction of ionizing radiation with melanin is poorly understood and has rarely been addressed in the literature. As melanin has potential usefulness for the design of new, nature-inspired radio-protective materials for a wide range of applications – from treatment and protection of cancer patients during radiation therapy to nuclear energy technology and space exploration – our analysis provides important new insights. Some of us recently reported on the remarkable stability of dry melanins towards ionizing radiation doses of 300 Gy [12]. To explain this phenomenon, it was suggested that the melanin polymer, studied in the form of dry *C. neoformans* cell ghosts, provides unique scattering and radical-scavenging properties. An investigation of the EPR properties and photoresponse of *C. neoformans* melanin in viable cells, including its response to γ-irradiation, was pursued here. Importantly, the high radiation doses applied are known to be more lethal to non-melanized cells, and thus explaining the role of melanin in protection of fungi was worthwhile investigating in more detail.

Our results demonstrated that EPR was useful to reveal alteration in melanin structure only under conditions where damage was enhanced, while irradiated cells were frozen in the presence of the radicals produced during irradiation. The intrinsic radical population in melanized *C. neoformans* cells under “resting” conditions is governed by Eq. 1 [15], [16] yet only a very small fraction of the melanin subunits exist in the semiquinone form. For example, quantitative EPR of a variety of melanins shows that the concentration of radicals (approximately 10^18^ spins/g) is accounted for by <0.1% of the subunits bearing a semiquinone [12]. The photoinduction process, while it increases radical population according to the reactions in Scheme 1, still reports on the properties of a small fraction of melanin. Charge transfer reactions between hydroquinone and quinone in the triplet state of illuminated melanin yield two semiquinone radicals (HQ^−**•**^) by comproportionation [42]. 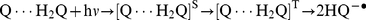
(Scheme 1)Our results show that a chemical analysis was more informative than EPR spectroscopy even when combined with illumination to probe melanin integrity, at least in the case of the physiologically relevant room temperature irradiations. More extensive analyses are required for a complete description of the phenomena and to reveal any mechanistic coupling between radical behavior and chemical reactivity. It is very clear that hydroxyl radical attack on susceptible sites in melanin subunits leads to C-C bond cleavage and the release of low molecular weight aldehydes.

Since melanized cells are not impervious to the effects of ionizing radiation in solution, a new question arises: Is melanin a sacrificial component of the cell surface architecture? The avirulent, acapsular *C. neoformans* cells used here lack an exterior polysaccharide coat but have an intact cell wall and cell membranes and, when melanized, contain an array of melanin particles in porous concentric layers within the cell wall [43]. A dense arrangement of these particles near the cell surface affords a protective physical barrier and was proposed to provide radiation protection by a combination of Compton scattering, energy attenuation of photons, and quenching of free electrons and free radicals generated by radiolysis of water [8], [12]. Most importantly, modifications of melanin structure can be metabolically repaired in viable fungal cells [43]. This supports the idea that melanin is a protective yet partially sacrificial cell wall component that may be damaged but still prevent cell death.

It is now clear that ionizing radiation leads to partial melanin damage/fragmentation in aqueous environments, which produces TBA-reactive substances related to those produced from other cellular components. A thorough understanding of the chemistry of melanin interactions with hydroxyl radical would help explain radiation resistance as a biological function of melanin. Sacrificial degradation of melanin should be included as a factor contributing to its radioprotective properties in cells.

## Supporting Information

Figure S1 **Optical spectra of melanin oxidation products.** A) supernatants collected from peroxide-treated melanized-cell ghosts and synthetic melanin; B) PDCA and PTCA synthesized from indole precursors, according to [12], [24].
(TIF)
