# Supplementary figures and images for: Protection of Melanized Cryptococcus neoformans from Lethal Dose Gamma Irradiation Involves Changes in Melanin's Chemical Structure and Paramagnetism
Source: PLoS One. 2011 Sep 22;6(9):e25092. doi: 10.1371/journal.pone.0025092 (PMC3178601; doi:10.1371/journal.pone.0025092)

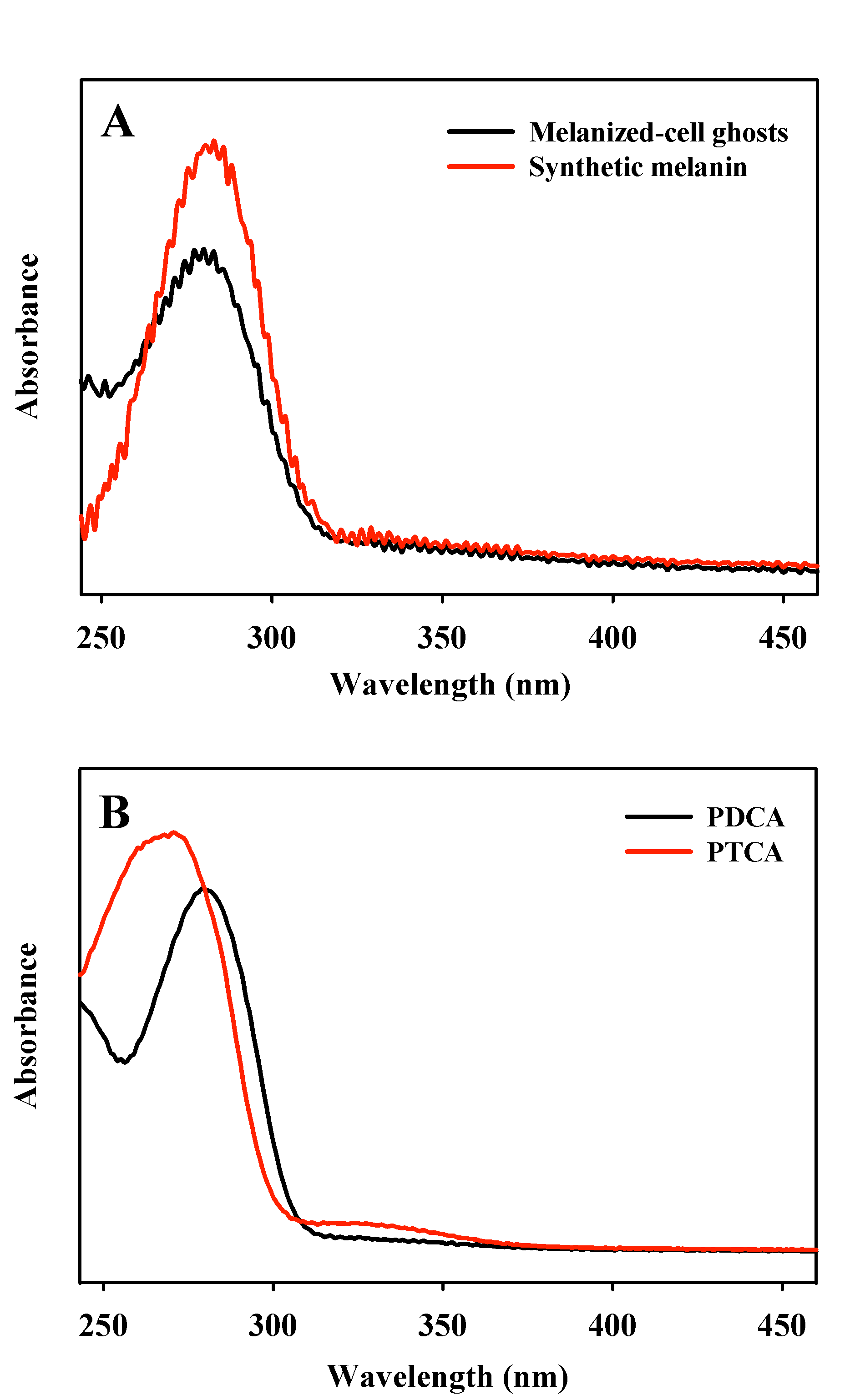

Supplement: Figure S1 — Optical spectra of melanin oxidation products. A) supernatants collected from peroxide-treated melanized-cell ghosts and synthetic melanin; B) PDCA and PTCA synthesized from indole precursors, according to [12], [24]. (TIF) [file pone.0025092.s001.tif]
